# Prophylactic Systemic Antibiotic and Systemic Glucocorticoid Therapy After Burn Inhalation Injury: A Report of Two Cases and Review of Literature

**DOI:** 10.7759/cureus.68285

**Published:** 2024-08-31

**Authors:** Huifen Lu, Jiayi Li, Guoli Quan, Haiyan Cui

**Affiliations:** 1 Respiratory and Critical Care Medicine, The Third Affiliated Hospital of Southern Medical University, Guangzhou, CHN

**Keywords:** treatment, antibiotics, glucocorticoids, inhalation injury, burn

## Abstract

Burn inhalation injury is a significant risk factor for mortality in burn patients. Despite the considerable progress made in the treatment of burn inhalation injury, there remains no consensus on the appropriate course of treatment, leading to ongoing controversy regarding the use of prophylactic systemic antibiotics and systemic glucocorticoids. This study presents two cases of burn inhalation injury diagnosed by fiberoptic bronchoscopy and treated with systemic glucocorticoids and prophylactic systemic antibiotics. By conducting a literature review, this study aimed to discuss the application of systemic glucocorticoids and prophylactic systemic antibiotics in patients with burn inhalation injuries. The suitability of prophylactic systemic antibiotics and systemic glucocorticoids for treating burn inhalation injury patients necessitates a comprehensive assessment of the patient's condition and an accurate judgment of the course of their disease.

## Introduction

Burns pose a significant global public health challenge. According to the World Health Organization, 180,000 people die from burns annually worldwide, leading to a substantial economic burden [1]. Burn inhalation injury is a major complication of burns and a primary cause of mortality among burn patients [1]. The mortality rate for burn patients is 10.9%, with an incidence of inhalation injury in hospitalized burn patients at 15.7% [2]. Burn inhalation injury occurs as a result of inhaling heat, toxic or irritating gases, and chemical irritants, leading to damage to the airway and lung tissue as well as systemic poisoning. Fiberoptic bronchoscopy allows direct observation of airway damage and enables diagnosis based on signs such as airway hyperemia, edema, carbon deposits, mucosal exfoliation erosion, and increased secretion. Burn inhalation injury can result in respiratory cilia damage, shedding of epithelial cells, excessive inflammatory secretions, severe bronchospasm, alveolar damage, and even acute respiratory distress syndrome (ARDS). The severity of burn inhalation injury is closely associated with an increased risk of mortality [3]. Burn inhalation injury can lead to complications such as respiratory obstruction, respiratory failure, and pulmonary infection, all of which contribute to a higher mortality rate among burn patients.

Nowadays, burn inhalation injury treatments include mucous dissolves, anticoagulation, bronchodilator therapy, bronchoscope lavage, airway management, respiratory support, fluid management, and prevention of pulmonary fibrosis. However, this article has shown two cases in which, after multiple bronchial lavages and prophylactic systemic antibiotics, short-term systemic glucocorticoids and inhaled bronchodilators, and mucus solutes were administered, two patients' symptoms improved significantly. The use of prophylactic systemic antibiotics and systemic glucocorticoids remains controversial. Prophylactic systemic antibiotics refer to the use of antibiotics orally or intravenously in the absence of evidence of infection. It can control respiratory and systemic infections in patients, reducing the mortality rate of critically ill patients. However, it may not have an impact on reducing the incidence and mortality rate of infections. Systemic corticosteroids refer to the use of corticosteroids orally or intravenously. It can suppress the production of inflammatory factors, thereby inhibiting the body's inflammatory response, reducing the occurrence of pulmonary infections and stress ulcers, shortening hospital stays, alleviating edema in important organs such as the lungs and brain, suppressing bronchospasm, preventing pulmonary fibrosis, and lowering coagulopathy and mortality rates. However, it may exacerbate existing infections and increase the risk of infection without benefiting lung function recovery or reducing common adverse reactions. Based on the treatment of these two cases of burn inhalation injury, this article discusses the aforementioned controversial treatments.

## Case presentation

On April 24 (day 0), 2024, two 18-year-old male students were caught in a fire in their dormitory and were trapped in the fire for more than 10 minutes. They were then taken to hospital by ambulance.

Case 1

He presented with dyspnea and chest tightness, accompanied by a pronounced burning sensation in the pharynx, difficulty swallowing, and cough with a large amount of smoky gray sputum. Additionally, he had burns on the face and upper limbs covering less than 10% of the total body surface area (TBSA) with a first-degree depth. His vital signs and lung auscultation were normal. He had a history of smoking one pack a day for two years but has since quit. Upon arrival at the emergency department, it was preliminarily determined that he may have suffered from a burn inhalation injury. Subsequently, he received 10 mg of intravenous dexamethasone, inhaled budesonide and ipratropium bromide, intravenous doxofylline, and wound disinfection. Although his pharyngeal burning and chest tightness had somewhat resolved, he continued to produce copious amounts of smoke-gray sputum. Therefore, he was transferred to the respiratory unit on day 1.

**Figure 1:**
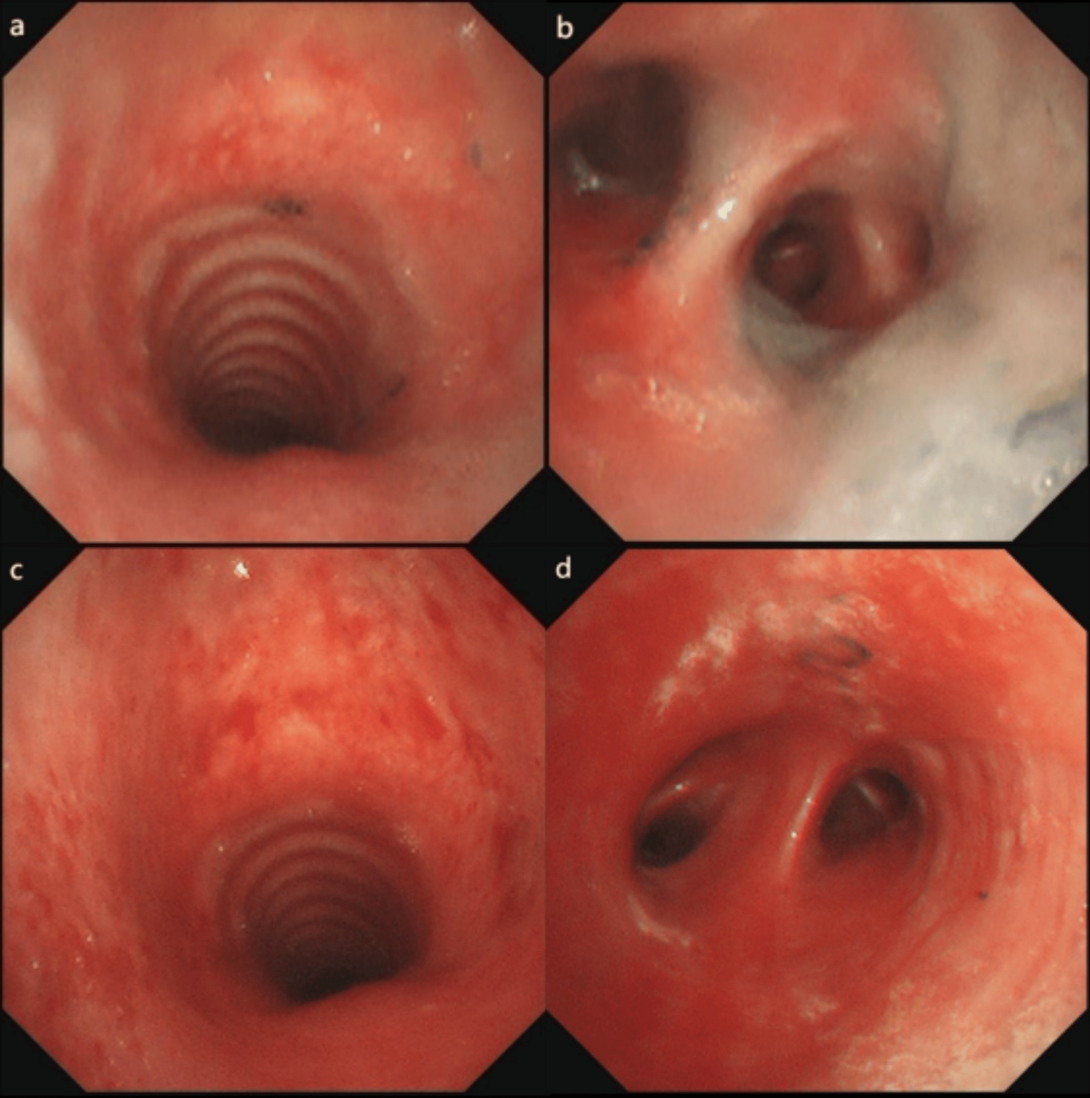
Bronchoscopy of Case 1 Examination with bronchoscopy in Case 1 revealed a gradual reduction of tracheal epithelial mucosal erosion and inflammation edema, with gradual clearance of black carbonaceous deposits. a and b were obtained on day 2. a: The upper segment of the trachea. b: The opening of the right lower lobe. c and d were obtained on day 4. c: The trachea. d: The opening of the right middle lobe.

Subsequently, pulmonary function tests indicated that the patient had moderate obstructive pulmonary ventilation dysfunction (Table 1). The level of *Mycoplasma pneumoniae* IgM antibody was 29.92 AU/mL. Blood gas analysis, blood routine, C-reactive protein (CRP), blood sedimentation (ESR), liver function, renal function, electrolyte levels, and blood coagulation function were within normal ranges. There were no apparent abnormalities found in the chest CT scan. On day 2, bronchoscopy revealed mild erosion of the epithelial tracheal mucosa and inflammation of the tracheal and bronchial mucosa with retention of large amounts of black carbon dust (Figure 1). The patient underwent repeated bronchial lavage and a second bronchoscopy on day 4. It showed semi-quantitative CAP1+ for *Haemophilus influenzae* and semi-quantitative BAP3+ for *Streptococcus pneumoniae* in fluid from bronchoalveolar lavage (BALF) culture. Respiratory pathogen targeted sequencing (tNGS) of BALF showed sequence numbers indicating *Mycoplasma pneumoniae* at 14,291 counts, *Haemophilus influenzae* at 10,362 counts, and *Streptococcus pneumoniae* at 26,315 counts. The final diagnosis included moderate burn inhalation injury, upper respiratory tract irritation syndrome, acute secondary laryngitis, acute secondary tracheobronchitis, and *Mycoplasma pneumoniae* infection. Treatment included daily oral administration of moxifloxacin (4 g), intravenous methylprednisolone (40 mg), inhaled oxygen with budesonide, and regular wound sterilization. With gradual improvement in symptoms, the methylprednisolone dose was reduced to 24 mg per day on day 4. On day 6, his symptoms had significantly improved, leading to his discharge from the hospital. Moxifloxacin, montelukast sodium, and eucalyptol-limonene-pinene enteric capsules were orally administered at home for a duration of seven days.

**Table 1:** Pulmonary function test The post-drug value refers to the measurement of lung function taken 20 minutes after inhaling 400 ug ventolin. The symbol "-" indicates that there is no available data for analysis. FEV: forced expiratory volume, FVC: forced vital capacity, MMEF: mid-maximum expiratory flow, MEF: maximum expiratory flow, MVV: maximum voluntary ventilation

Terms	Case 1					Case 2				
	Prediction	Pre-drug	Pre-drug/prediction	Post-drug	Post-drug/prediction	Prediction	Pre-drug	Pre-drug/prediction	Post-drug	Post-drug/prediction
FEV1 (L)	3.79	1.99	65.96	1.99	-	4.33	2.90	66.97	3.09	71.36
FVC (L)	4.26	2.81	65.96	2.81	-	4.93	4.02	81.54	4.16	84.38
FEV1/FVC (%)	88	71	80.68	71	-	87.26	72.14	82.67	74.28	85.12
MMEF75/25 (L/s)	4.16	1.35	32.45	1.35	-	4.62	2.18	47.19	2.46	53.25
MEF50 (L/s)	5.32	1.53	28.76	1.53	-	5.69	2.45	43.06	2.75	48.33
MEF25 (L/s)	7.99	3.70	46.31	3.70	-	8.53	4.38	51.35	4.78	56.04
MVV (L/min)	143.58	-	-	-	-	155.40	-	-	-	-

Case 2

He presented with a cough and profuse production of smoke-gray sputum, accompanied by dyspnea, shortness of breath, hoarseness, and difficulty swallowing. Additionally, he had burns on both upper limbs of less than 10% of TBSA with a first-degree depth. His vital signs and lung auscultation were normal, and he had no significant medical history. It was preliminarily determined that he may have suffered from burn inhalation injury. In the emergency department, he received treatment including inhaled budesonide and acetylcysteine, as well as wound disinfection. Although his voice became less hoarse and his shortness of breath improved slightly, he continued to produce a large amount of soot sputum. As a result, he was referred to the respiratory department the following day.

**Figure 2:**
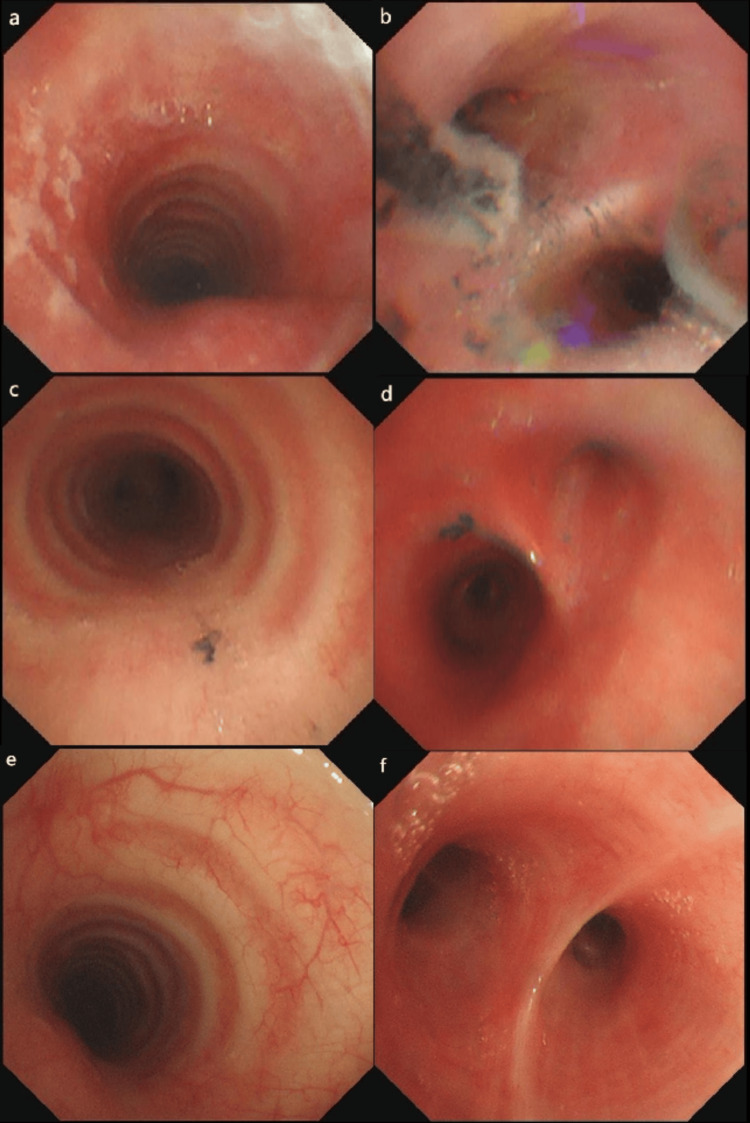
Bronchoscopy of Case 2 The tracheoscopy examination of Case 2 revealed a gradual reduction in tracheal epithelial mucosal erosion and inflammatory edema, with the gradual clearance of black carbonaceous deposits. a and b were obtained on day 2. a: The upper segment of the trachea. b: The posterior segment of the right upper lobe of the lung. c and d were obtained on day 4. c: The upper segment of the trachea. d: The right upper lobe and right middle bronchus of the lung. e and f were obtained on day 12. e: The upper segment of the trachea. f: An opening in the right upper lobe of the lung.

Subsequent pulmonary function tests revealed moderate to severe mixed pulmonary ventilation dysfunction (Table 1). Blood gas analysis, blood routine, CRP levels, coagulation function, liver function, renal function, and electrolytes all fell within normal ranges. There were no apparent abnormalities found in the chest CT scan. On day 2, he underwent his first bronchoscopy, which revealed mild erosion of the epithelial tracheal mucosa, inflammation of the tracheal and bronchial mucosa, and retention of large amounts of black carbon dust, and was treated with repeated bronchial lavage (Figure 2). A second and third bronchoscopy were conducted on days 4 and 12, respectively. The results of BALF culture indicated that the presence of *Streptococcus viridans* was BAP semi-quantitative 3+, *Neisseria* was BAP semi-quantitative 1+, and coagulase-negative G+ *Staphylococcus* was BAP semi-quantitative 2+. The tNGS in BALF revealed sequence numbers for *Haemophilus influenzae*, *Fusobacterium nucleatum*, and influenza A virus as 22712, 876, and 9565, respectively. The patient was ultimately diagnosed with moderate burn inhalation injury, upper respiratory tract irritation syndrome, acute secondary laryngitis, and acute secondary tracheobronchitis. Treatment included daily intravenous cefmetazole 1 g, intravenous methylprednisolone 40 mg, and aerosol inhalation of terbutaline, ipratropium bromide, budesonide, and acetylcysteine. Regular wound disinfection was also administered. With gradual improvement in symptoms, methylprednisolone was discontinued on day 9. Finally, all medications were stopped on day 13, and the patient was discharged from the hospital.

## Discussion

The most common complication of burn inhalation injury is respiratory infection, which is attributed to the disruption of airway wall integrity caused by shedding of the airway epithelium and impaired airway clearance due to damaged cilia. In a study conducted by Shirani et al., 1,058 burn patients at a single institution were evaluated, among whom 35% were diagnosed with inhalation injuries. Of these patients, 38% developed pneumonia within seven to eight days after the burn injury [4]. The mortality rate for burn patients increases by up to 20% with inhalation injury alone, up to 40% with pneumonia alone, and up to approximately 60% when both complications coexist. These findings indicate that inhalation injury and pneumonia have significant, independent, and additive effects on burn mortality [4]. Although inflammatory infiltrates were initially absent on chest CT scans, pathogens were identified in FBAL within 24 hours after the burn injury in two cases, leading to a diagnosis of tracheobronchial infection. Pneumonia did not develop in these two burn inhalation injury patients after receiving prophylactic systemic antibiotics. However, the use of prophylactic systemic antibiotics remains quite controversial.

There is a study suggesting that prophylactic systemic antibiotics are inappropriate after burn inhalation injury [5]. Currently, the study of prophylactic systemic antibiotics after burn inhalation injury is primarily focused on patients with severe burn inhalation injury and/or large-area burns. A retrospective study of 58 burn patients with inhalation injuries indicates that prophylactic systemic antibiotics do not have a statistically significant effect on the development of pneumonia [6]. A retrospective study of sepsis in 147 burn patients over a four-year period revealed that prophylactic systemic antibiotics had no impact on the occurrence and mortality of sepsis [7]. The use of broad-spectrum antibiotics in severe burn patients can lead to vitamin K deficiency-associated coagulopathy [8]. On the contrary, other studies suggest that an early and appropriate combination of broad-spectrum antibiotics plays a crucial role in controlling respiratory system and systemic infections in patients. This can lead to reduced mortality, especially in critically ill patients (such as those with severe inhalation injury, extensive severe burns, respiratory failure, shock, and immunocompromised patients) [6,9]. The use of prophylactic systemic antibiotics is controversial mainly due to the high mortality rate of critically ill patients, which may lead to an underestimation of their efficacy. However, there is a lack of studies on mild and moderate inhalation injuries. In this report, we present two cases of moderate burn inhalation injury that were treated with prophylactic systemic antibiotics and achieved good results. These findings suggest that prophylactic systemic antibiotics may be effective in patients with mild and moderate burn inhalation injuries, but the sample size is small, and further verification is needed.

Furthermore, the successful treatment of the two burn inhalation injury patients in this study was closely linked to the early administration of short-term systemic glucocorticoids (7 and 10 days, respectively), which significantly alleviated their irritating cough. Glucocorticoids have the ability to stabilize lysosomal function and reduce pulmonary fibrosis, as well as mitigate early pulmonary edema, cerebral edema, and severe bronchospasm in severe burn inhalation injury cases. Glucocorticoids can also stimulate alveolar type II cells to enhance the secretion of alveolar surfactant, which serves to protect the airways. Research has shown that glucocorticoids are able to counteract neurogenic plasma extravasation by increasing the expression of neutral endopeptidase (NEP) in human airway tissues. NEP functions to deactivate neuropeptides by cleaving them, thereby mitigating their effects [10]. Current research on systemic glucocorticoids following burn injury and its treatment has primarily been conducted in animal models, particularly rats. Studies involving human subjects have predominantly been retrospective in nature. Administration of low-dose glucocorticoids during the acute phase of burns covering more than 70% of body surface area has been shown to decrease levels of proinflammatory cytokines such as CRP, tumor necrosis factor-α, interleukin-6, and interleukin-8. This reduction in cytokine levels is associated with a decreased incidence of pulmonary infections and stress ulcers, as well as a shortened hospital stay for patients [11]. A moderate dose of methylprednisolone (4 mg/kg) has been shown to significantly reduce inflammation, coagulopathy, and mortality in the acute phase of treating acute lung injury caused by smoke inhalation in rats [12]. Administering large doses of glucocorticoids can effectively reduce pulmonary edema and associated mortality in mice with smoke inhalation injury [13]. Additionally, a three-day course of methylprednisolone treatment may be sufficient to alleviate advanced fibrotic changes in rats following smoke inhalation [12]. Early and continuous administration of glucocorticoids has been shown to significantly reduce the incidence of ARDS caused by inhalation of white smoke. Furthermore, sequential administration of glucocorticoids has demonstrated efficacy in treating patients with late-stage pulmonary fibrosis resulting from white smoke inhalation [14]. On the contrary, some studies suggest that systemic glucocorticoids are currently considered inappropriate [5,15]. Patients with large-area burns who undergo routine and long-term steroid treatment may experience an increased incidence of infection. A study on severe burn patients revealed that the use of steroids was linked to a 41% rise in mortality and a significant increase in infectious complications such as pneumonia and bacteremia. This correlation may be attributed to the larger burn area among patients receiving steroid treatment [16]. One study indicated that short-term steroid therapy did not have any impact on the ultimate recovery of lung function in patients with milder isolated smoke inhalation injuries [17]. Based on a study, it was found that exposure to smoke before receiving glucocorticoid treatment does not reduce the occurrence of common adverse reactions following smoke inhalation [18].

## Conclusions

In this paper, the treatment of two cases of burn inhalation injury is a significant challenge. Attention should be given not only to managing airway and lung injury but also to controlling complications such as infection and preventing airway stenosis. The use of prophylactic systemic antibiotics and systemic glucocorticoids in treating burn inhalation injury is controversial due to the increased risk of infection and associated mortality in critically ill patients with burn inhalation injury. Therefore, the following recommendations are provided for reference in dealing with different situations of burn inhalation injury encountered in clinical practice. Patients with severe burn inhalation injury and/or extensive burns complicated with respiratory failure and shock should receive early prophylactic systemic antibiotic treatment. For patients diagnosed only with severe burn inhalation injury and/or extensive burns, caution should be exercised when considering prophylactic systemic antibiotic therapy, requiring professional assessment. In cases of early burn inhalation injury complicated with pulmonary edema, acute lung injury, or ARDS, short-term use of systemic glucocorticoids can be considered. For late-stage pulmonary fibrosis in burn inhalation injury patients, short-term use of systemic glucocorticoids may be extended if initial effectiveness is not achieved. However, systemic glucocorticoids should be avoided in immunodeficient or extensively burned burn inhalation injury patients due to the increased risk of infection complications and associated mortality. The study of prophylactic systemic antibiotics and systemic corticosteroids in burn inhalation injury requires a large number of samples for randomized controlled trials in the research of different degrees of burn inhalation injury and different complications, especially in human studies.
